# Prognostic value of [^18^F]FDG PET/CT in metastatic hormone-sensitive prostate cancer at initial diagnosis: a retrospective cohort study

**DOI:** 10.1080/07853890.2024.2411017

**Published:** 2024-10-11

**Authors:** Ang Li, Kai Shen, Yiyi Ji, Weiwei Zhang, Bo Liu, Ruopeng Su, Xiang Zhou, Liang Dong, Yinjie Zhu, Baijun Dong, Jiahua Pan, Qi Wang, Wei Xue

**Affiliations:** a Department of Urology, Ren Ji Hospital, Shanghai Jiao Tong University School of Medicine, Shanghai, China; b Department of Nuclear Medicine, Ren Ji Hospital, Shanghai Jiao Tong University School of Medicine, Shanghai, China; c Shanghai Key Laboratory for Tumor Microenvironment and Inflammation, Shanghai Jiao Tong University School of Medicine, Shanghai, China

**Keywords:** Metastatic hormone-sensitive prostate cancer, ^18^F-fludeoxyglucose, visceral metastasis, bone metastasis, prognosis

## Abstract

**Introduction:**

This retrospective study aimed to evaluate the prognostic value of [^18^F]FDG parameters in patients with visceral and bone metastatic hormone-sensitive prostate cancer (mHSPC).

**Patients and methods:**

This analysis included the mHSPC patients who underwent [^18^F]FDG PET/CT at the initial diagnosis. Baseline characteristics were analyzed, and the uptake of [^18^F]FDG was quantified using SUV_max_. Kaplan–Meier and Cox proportional hazard regression analysis were employed to evaluate the correlation between SUV_max_ and patient survival.

**Results:**

Among the 267 patients enrolled, 90 (33.7%) presented with visceral metastases and 177 (66.3%) had bone metastases. The median follow-up for the visceral metastasis group was 35.5 months (IQR 26–53.8 months). The median overall survival for patients with lung, liver, or both metastases were 30, 21 and 17 months, respectively. Patients exhibiting higher [^18^F]FDG uptake in metastatic lesions experienced shorter overall survival (OS) in comparison to those with lower [^18^F]FDG uptake, both in the visceral metastases group (17 vs. 31 months, *p* = 0.002) and the bone metastases group (27.5 vs. 34.5 months, *p* < 0.001). Cox regression analysis further revealed that increased [^18^F]FDG uptake in metastatic lesions emerged as a significant risk factor in both OS and progression-free survival (PFS). In contrast, the variability in [^18^F]FDG uptake in primary lesions did not provide a reliable indicator for predicting prognosis.

**Conclusions:**

In mHSPC patients, higher [^18^F]FDG uptake in metastatic lesions indicates shorter survival and increased risk of disease progression. The [^18^F]FDG SUV_max_ in primary tumors did not show significant prognostic value. Our study underscores the unique prognostic potential of [^18^F]FDG PET/CT in mHSPC patients, highlighting its importance in the management of both bone and visceral metastases.

## Introduction

According to recent data from the American Cancer Society, prostate cancer stands as the second most prevalent cancer afflicting men globally [1]. While the majority of these cases are initially diagnosed with localized prostate cancer with a favorable 5-year relative survival rate exceeding 98% post radical prostatectomy or androgen deprivation therapy (ADT) [2], there has been a notable increase in the incidence of metastatic hormone-sensitive prostate cancer (mHSPC) at first clinical presentation, rising from 0.58 to 2.74% over recent years [3,4]. More troubling is that mHSPC patients face a significantly diminished 5-year cancer-specific survival rate, hovering around 30% [2]. While most distant metastases occur in bones and lymph nodes, previous studies have suggested that nearly 38% of newly-diagnosed mHSPC patients exhibit visceral metastases [5]. Compared with lymph node or bone metastasis, visceral metastasis in mHSPC led to a significantly greater risk of overall and cancer-specific mortality [6,7].

Molecular imaging techniques, notably positron emission tomography (PET) and its hybrid counterparts, like PET/CT and PET/MR, have played a crucial role in mHSPC diagnosis [8]. One of the common nuclear tracers,^18^F-fludeoxyglucose ([^18^F]FDG), has been widely used to evaluate metastasis, predict therapeutic efficacy, and assess prognosis [9]. Although traditionally [^18^F]FDG PET/CT has limited diagnostic value for primary prostate cancer, recent studies have demonstrated its advantages in evaluating disease progression and detecting metastatic lesions in patients with advanced prostate cancer [10–13]. Consequently, we aim to explore the potential benefits of [^18^F]FDG PET/CT in managing metastatic prostate cancer.

Building on prior research, this study provided a comprehensive analysis of the clinical characteristics and investigated the prognostic implications of [^18^F]FDG PET/CT parameters in patients with mHSPC. Based on these clinical factors, we emphasized the prognostic value of [^18^F]FDG PET/CT parameters in both overall survival (OS) and progression-free survival (PFS) in mHSPC. Our analysis may provide insights into the clinical utility of [^18^F]FDG PET/CT in mHSPC management.

## Patients and methods

### Patients selection and examination

This retrospective study was performed in line with the principles of the 1975 Declaration of Helsinki. Approval was granted by the Shanghai Jiao Tong University School of Medicine, Shanghai Ren Ji Hospital Ethics Committee (RA-2021-242). The study received a waiver of informed consent from the Shanghai Jiao Tong University School of Medicine, Ren Ji Hospital Ethics Committee, due to the retrospective nature of the study and the use of anonymized clinical data. In this study, we identified patients with bone or visceral metastatic hormone-sensitive prostate cancer from a total of 2736 patients who completed [^18^F]FDG PET/CT scans. These patients had their initial clinical evaluations at Shanghai Ren Ji Hospital between 2013 and 2021. Experienced urologists recommended them to receive a [^18^F]FDG PET/CT scan based on their high tumor burden, leading to the diagnosis of distant metastases. We included these patients in our study unless they met the following exclusion criteria:

1. Clinical manifestations of castration resistance, evidenced by newly-detected PSA elevations, local recurrences, or distant metastases post anti-androgen therapy, radical surgery, or radiotherapy.
2. Inability to furnish comprehensive prognostic data, encompassing details about treatments received or the duration of survival.
3. Loss to subsequent follow-up.

Given the constrained application of biopsies for metastatic lesions in prostate cancer, our primary reliance was on PET/CT scan results to detect most visceral and bone metastases. Distant metastases, encompassing both bone and visceral metastases, were predominantly pinpointed using computed tomography (CT) images, interpreted by seasoned nuclear radiologists. Confirmation of metastasis was further substantiated by heightened [^18^F]FDG uptake in the areas of suspicion.

We categorized patients with visceral metastases into three groups based on the metastatic lesion sites: those with lung metastases, liver metastases, and a combination of both lung and liver metastases. However, we excluded patients with metastases in other regions, such as the brain or pancreas, due to the limited sample size for these particular cases.

### PET/CT protocol and image analysis

For our study, all selected patients underwent an [^18^F]FDG PET/CT scan at Shanghai Ren Ji Hospital during their initial diagnosis. These scans were executed using a multislice PET/CT camera, adhering to established clinical protocols. Prior to the [^18^F]FDG injection:

1. Patients were instructed to fast for a minimum of 6 hours.
2. Their blood glucose levels were assessed, ensuring they were below 150mg/dL.
3. Each patient was administered an intravenous injection equating to roughly 7.4 MBq of [^18^F]FDG per kilogram of their body weight. Following this injection, patients rested for an hour before undergoing the PET/CT imaging process.

PET and non-contrast CT images were acquired, reconstructed and analyzed on a Siemens Healthineers AG scanner (Erlangen, Germany). Lesions manifesting the highest [^18^F]FDG uptake were chosen as target lesions for metastasis evaluation. A pair of seasoned nuclear medicine physicians reviewed the PET/CT images. The maximum SUV (SUV_max_) values, adjusted for body weight (BW), were determined using the following equation: SUV_max_ = Injected Dose (MBq)/Body Weight (g).

### Outcomes

For all the patients included in this study, we collected and examined baseline characteristics such as age, clinical TNM stage, Gleason score, serum PSA level, and [^18^F]FDG PET/CT parameters. To compare prognoses based on [^18^F]FDG uptake levels, we used the median of SUV_max_ as the cutoff value in each group to stratify patients into high and low uptake subgroups. Our study primarily focused on the overall survival (OS) metric, which was defined as the duration between the initial PET/CT scan and the date of death. The secondary outcome was progression-free survival (PFS), characterized as the time elapsed from the initial PET/CT scan up to either disease progression or death from any etiology. Typical endpoint events incorporated within the PFS analysis included:

1. Castration-resistant manifestations: Including consecutive increases in PSA levels on at least 3 occasions, and/or radiographic progression defined as ‘Progressive Disease (PD)’ according to RECIST 1.1 criteria [14].
2. New distant metastatic lesions identified by routine PET/CT or PET/MR scan.
3. Death.

Follow-ups continued until November 2022. For patients who began follow-up before 2017, we conducted a retrospective observation of all the patients for up to 60 months, during which we monitored tumor progression through clinical visits or telephone follow-ups. For patients lost to follow-up, we employed the Last Observation Carried Forward (LOCF) method to handle missing data, ensuring continuity and completeness of the study data.

### Statistical analysis

To determine differences among continuous ­variables, we employed one-way ANOVA for data adhering to a normal distribution, and the Kruskal–Wallis test was used for data deviating from ­normality. Categorical variables were subjected to the Chi-square test for analysis. For evaluating survival rates, both Kaplan–Meier and Cox proportional risk regression analyses were utilized. In all analyses conducted, a *p*-value of less than 0.05 was deemed statistically significant. Statistical computations and graphical representations were executed using *IBM SPSS version 26.0* and *GraphPad Prism 9*, respectively.

## Results

### Characteristics of patients

**Figure 1.**
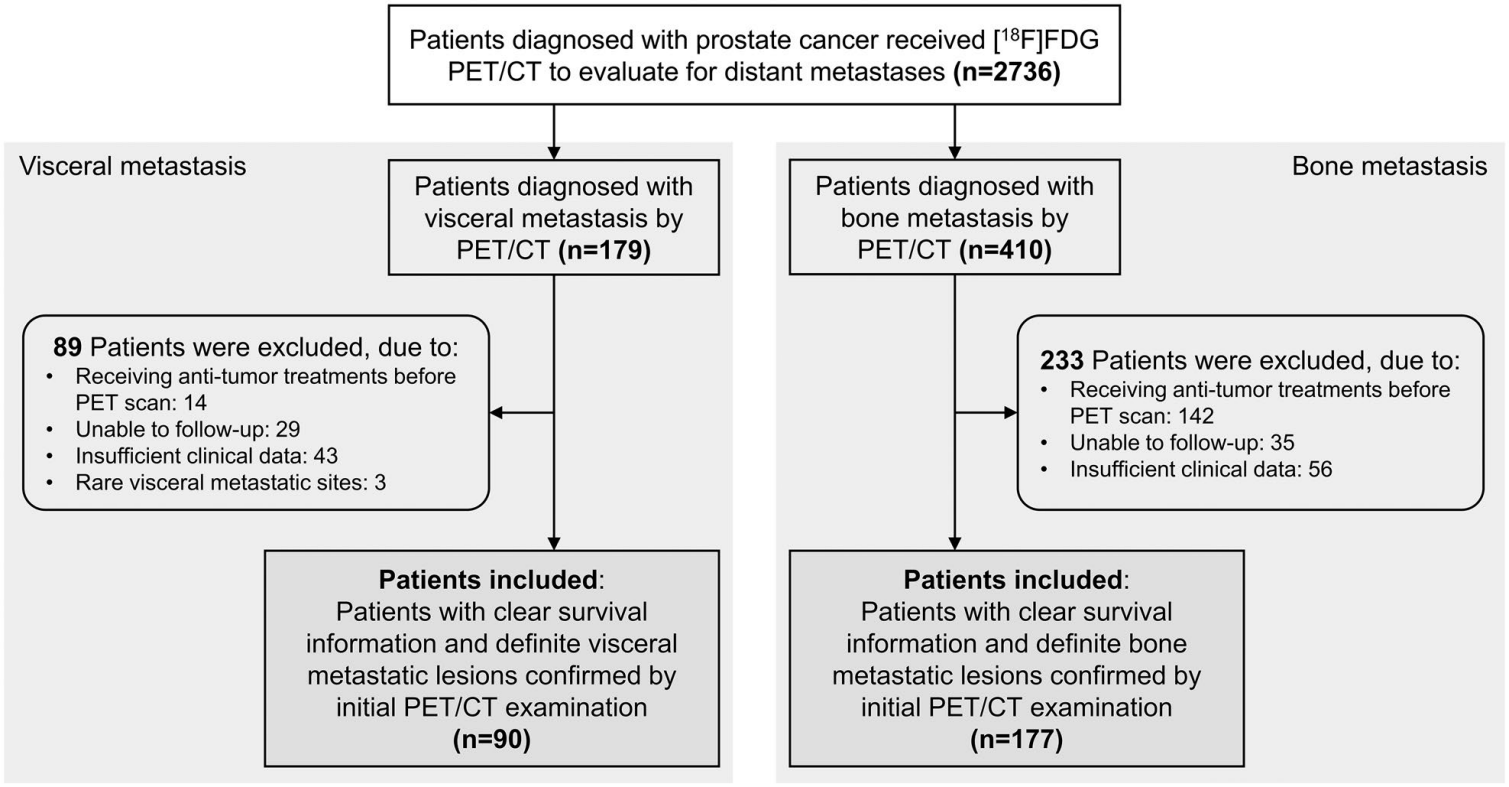
Flow diagram of included and excluded patients in this study.

**Figure 2.**
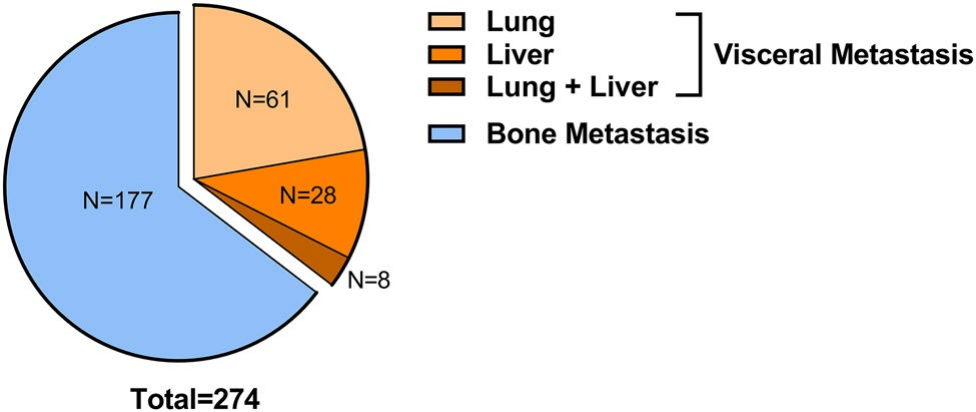
Constitution of the mHSPC cohort.

Our cohort initially consisted of 447 patients diagnosed with metastatic prostate cancer *via* [^18^F]FDG PET/CT. Following eligibility screening, a subset of 267 patients met the inclusion criteria for our retrospective study (Figure 1). Of this subset, 90 patients (33.7%) manifested with visceral metastases, while the remaining 177 patients (66.3%) had bone metastases. All distant metastases were pinpointed via [^18^F]FDG PET/CT during their preliminary clinical encounter. Breaking down the visceral metastases cohort further, we found that 61 patients (67.8%) had lung metastases, 21 patients (23.3%) had liver metastases, and 8 patients (8.9%) presented with metastases in both the lung and liver (Figure 2).

The baseline characteristics of these patients were elaborated in Tables 1 and 2. Patients with visceral metastases had a notably higher proportion of advanced clinical T stage (*T* ≥ 3) than those with bone metastases (67.8 versus 41.3%, *p* < 0.0001). Nevertheless, no discernible statistical variance was observed in clinical characteristics amongst patients categorized by different visceral metastatic sites (Table 2).

**Table 1.** Baseline characteristics of patients with initially diagnosed metastatic prostate cancer with bone and visceral metastasis included in the study.

	Visceral metastasis	Bone metastasis	*p*-Value
Number of patients, *n* (%)	90 (33.7)	177 (66.3)	
Median age (IQR[1])	71 (66-77)	72 (66-77)	0.31
Clinical T stage			***<0.001
T = 2, *n* (%)	29 (32.2)	104 (58.7)	
T = 3, *n* (%)	25 (27.8)	47 (26.6)	
T = 4, *n* (%)	36 (40.0)	26 (14.7)	
Clinical *N* stage			0.13
*N* = 0, *n* (%)	24 (26.7)	65 (36.7)	
*N* = 1, *n* (%)	66 (73.3)	112 (63.3)	
Gleason score			0.09
6-7, *n* (%)	15 (16.7)	50 (28.3)	
8, *n* (%)	25 (27.8)	51 (28.8)	
9-10, *n* (%)	22 (24.4)	31 (17.5)	
Data missing, *n* (%)	28 (31.1)	45 (25.4)	
Serum PSA level			0.46
Median (IQR)	79.62 (26.13 - 155)	63.61 (15.64 - 154)	
Data missing, *n* (%)	15 (16.7)	35 (19.8)	

[1] IQR: interquartile range.

[2] Significance level set at p < 0.05. *p < 0.05, **p < 0.01, ***p < 0.001.

**Table 2.** Baseline characteristics of patients with initially diagnosed metastatic prostate cancer with visceral metastasis.

	Lung metastasis	Liver metastasis	Lung + liver metastasis	*p*-Value
Number of patients, *n*(%)	61 (67.8)	21 (23.3)	8 (8.9)	
Median age (IQR[1])	70 (65-76)	71 (67-77)	74 (68.8-81.8)	0.13
Clinical T stage				0.56
T = 2, *n* (%)	18 (29.5)	9 (42.9)	2 (25.0)	
T = 3, *n* (%)	19 (31.2)	3 (14.3)	3 (37.5)	
T = 4, *n* (%)	24 (39.3)	9 (42.9)	3 (37.5)	
Clinical *N* stage				0.25
*N* = 0, *n* (%)	13 (21.3)	8 (38.1)	3 (37.5)	
*N* = 1, *n* (%)	48 (78.7)	13 (61.9)	5 (62.5)	
Gleason score				0.62
6-7, *n* (%)	8 (13.1)	5 (23.8)	2 (25.0)	
8, *n* (%)	18 (29.5)	5 (23.8)	2 (25.0)	
9-10, *n* (%)	17 (27.9)	4 (19.0)	1 (12.5)	
Data missing, *n* (%)	18 (29.5)	7 (33.3)	3 (37.5)	
Serum PSA level				0.65
Median (IQR)	85.92 (23.07 − 154.75)	94.65 (34.18 - 155)	43.6 (32.23 − 88.09)	
Data missing, *n* (%)	11 (18.0)	3 (14.3)	1 (12.5)	

[1] IQR: interquartile range.

Supplementary Table 1 provides a detailed summary of the treatments at the beginning of follow-up and after disease progression in both the visceral and bone metastases groups. Compared with the visceral metastases group, 21 (11.9%) patients with bone metastases had undergone radical prostatectomy by the time of our last follow-up. Before the surgery, all these patients received neoadjuvant therapies, including androgen deprivation therapy or chemotherapy. The variations of treatment reflect the personalized management strategies for different patient groups, which could potentially affect survival outcomes.

### Follow-up outcomes and survival analyses

**Figure 3.**
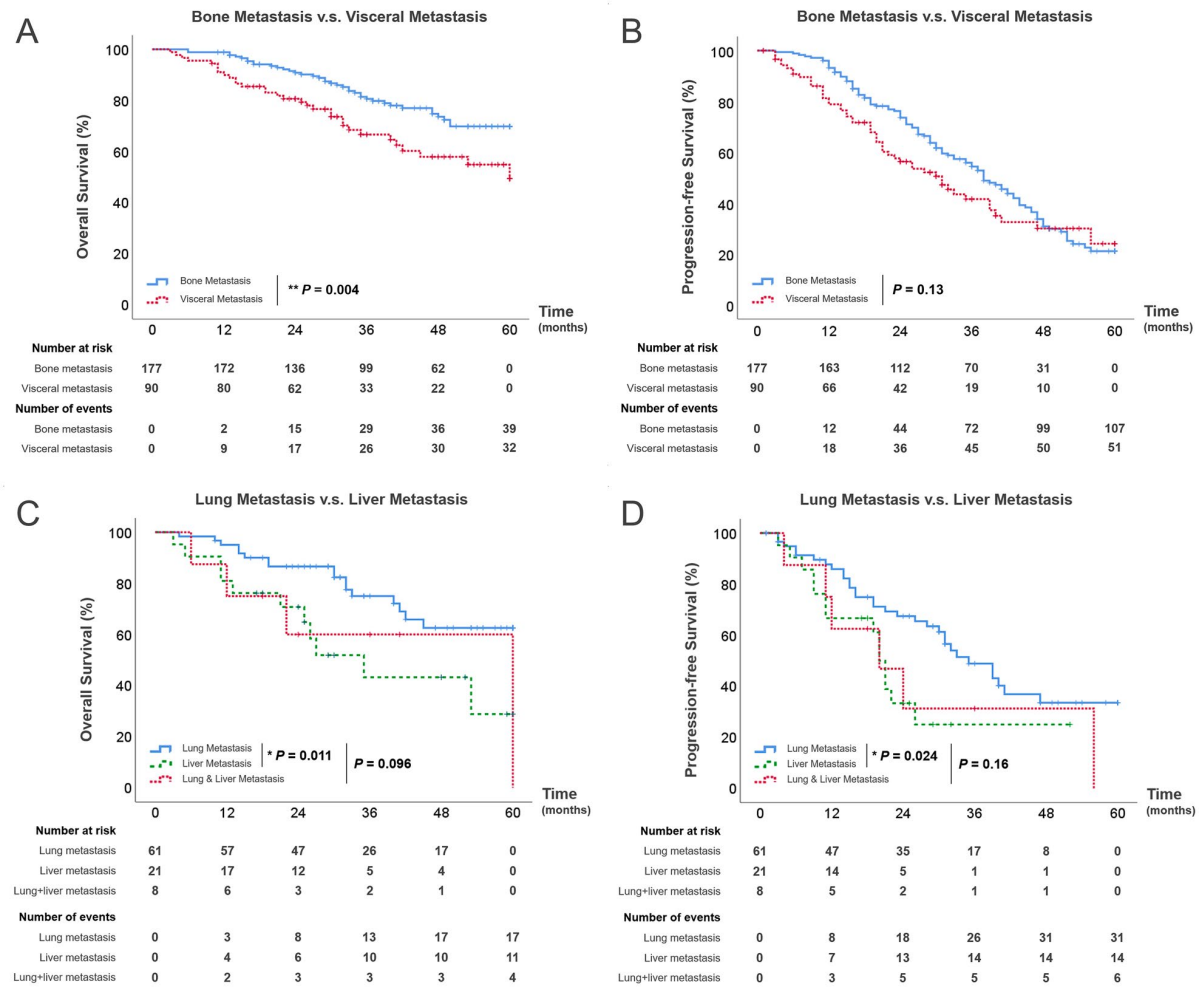
(A) Kaplan–Meier survival curves for OS among patients with visceral metastasis and bone metastasis; (B) Kaplan–Meier survival curves for PFS between patients with visceral metastasis and bone metastasis; (C) Kaplan–Meier survival curves for OS among mHSPC patients according to the site of visceral metastasis; (D) Kaplan–Meier survival curves for PFS among metastatic mHSPC patients according to the site of visceral metastasis.

The median follow-up period for patients with visceral metastases was established at 30.5 months (IQR 22–47.8 months), while for those with bone metastases, it was 39 months (IQR 27-59 months). By the end of this period, in the visceral metastases group, 32 patients (35.5%) had passed away, 50 patients (55.6%) remained alive, and 8 patients (8.9%) were no longer available for follow-up. In the bone metastases group, 36 patients (20.3%) had passed away, 128 patients (72.3%) remained alive, and 13 patients (7.3%) were no longer available for follow-up. Furthermore, 51 patients (56.7%) in the visceral metastases group encountered disease progression by the end of this period, compared to 107 patients (60.5%) in the bone metastases group. As shown in Figure 3(A,B), the median OS and PFS from the moment of visceral metastasis diagnosis until the endpoints were 21.5 months (IQR 11.8–32.3 months) and 19 months (IQR 11–27 months), respectively. For patients with bone metastases, the median OS and PFS were 29 months (19.3–35.3 months) and 27 months (IQR 17–39.5 months), respectively. The leading endpoints for PFS among patients who experienced disease progression included castration resistance (*N* = 74, 46.8%), new distant metastasis emergence (*N* = 54, 34.2%), and death (*N* = 30, 19.0%).

We further investigated the prognosis of mHSPC patients. We categorized mHSPC patients based on different visceral metastatic locations in relation to OS and PFS (Figure 3(C,D)). Patients with lung metastases showed a median OS of 30 months (IQR 14–33 months) and a median PFS of 19 months (IQR 13–31.5 months). Contrarily, patients with liver metastases were confronted with a significantly worse prognosis, with a median OS of 21 months (IQR 11–26.5 months, *p* = 0.011) and a median PFS of 15 months (IQR 9–20.8 months, *p* = 0.024). However, no significant variation in OS (*p* = 0.096) or PFS (*p* = 0.16) was observed when comparing patients solely presenting lung metastases to those with both lung and liver metastases.

**Figure 4.**
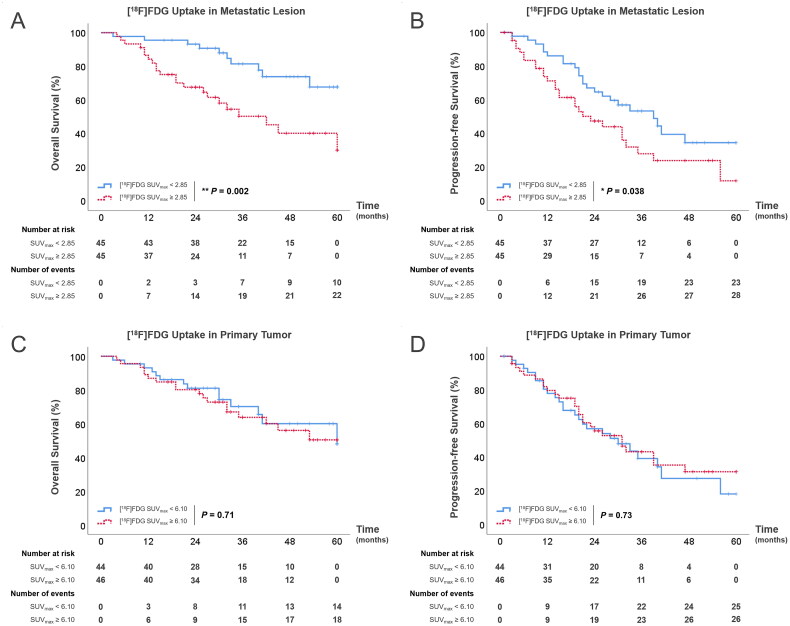
(A,B) Kaplan–Meier survival curves for OS (A) and PFS (B) among patients with visceral metastases according to [^18^F]FDG SUV_max_ of metastatic lesions; (C,D) Kaplan–Meier survival curves for OS (C) and PFS (D) among patients with visceral metastases according to [^18^F]FDG SUV_max_ of primary tumors.

**Figure 5.**
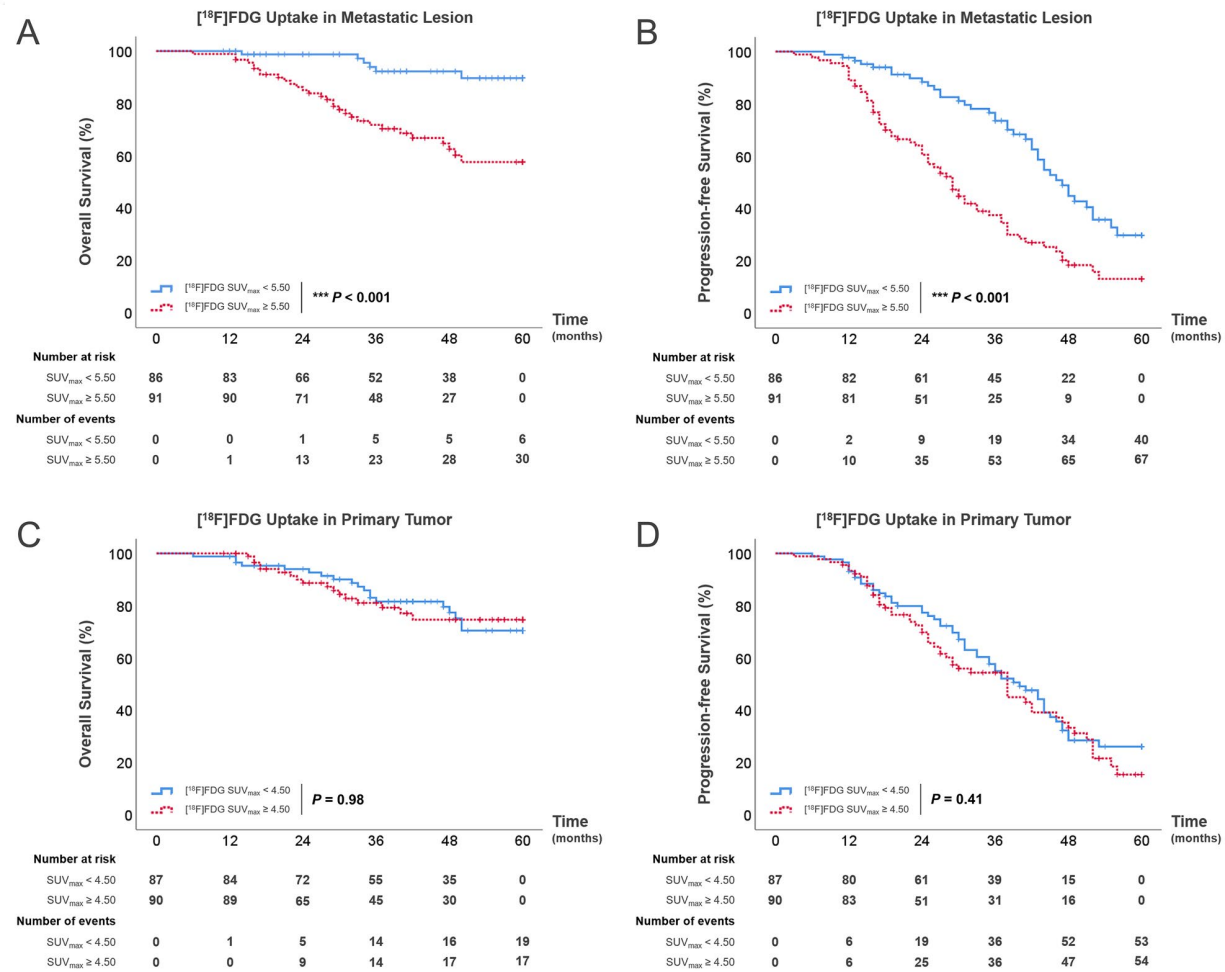
(A,B) Kaplan–Meier survival curves for OS (A) and PFS (B) among patients with bone metastases according to [^18^F]FDG SUV_max_ of metastatic lesions; (C,D) Kaplan–Meier survival curves for OS (C) and PFS (D) among patients with bone metastases according to [^18^F]FDG SUV_max_ of primary tumors.

Furthermore, we found the association between PET/CT parameters and prognosis, with a particular focus on the SUV_max_ of [^18^F]FDG within both the primary and metastatic lesions. In each patient group, we calculated the median SUV_max_ for both primary tumors and metastatic lesions independently, employing these medians as thresholds to delineate high and low uptake groups and explore differences in survival outcomes. In the visceral metastases group, patients with high [^18^F]FDG uptake in metastatic lesions (SUV_max_ ≥2.85) exhibited significantly poorer outcomes compared to those with low uptake (SUV_max_ <2.85) (Figure 4(A,B)). Specifically, the median OS for the high uptake group was 17 months (IQR 11.3–29.3 months), in contrast to the low uptake group, for which median OS was 31 months (IQR 22.8–38.3 months, *p* = 0.002). Similarly, the median PFS for patients with high [^18^F]FDG uptake was only 14.5 months (IQR 8.3–22.8 months), versus 21 months (IQR 13–29.5 months, *p* = 0.038) in the low uptake group. In the bone metastases group, although the overall prognosis is better than in the visceral metastases group, patients with high uptake in metastatic lesions (SUV_max_ ≥5.50) still had worse outcome compared with those with low uptake (Figure 5(A,B)). Median OS and PFS for the high uptake bone metastases group were 27.5 months (IQR 17.8–34.5 months) and 24 months (IQR 15.5–32 months), while patients with low [^18^F]FDG uptake (SUV_max_ <5.50) had a median OS of 34.5 months (IQR 33.3–35.8 months, *p* < 0.001) and a median PFS of 38 months (IQR 25.8–45.3 months, *p* < 0.001). Interestingly, patients with high uptake of [^18^F]FDG in primary tumors within both the visceral and bone metastases groups demonstrated no statistically significance in survival outcomes (Figures 4(C,D) and 5(C,D)), which elucidated the limited predictive value of primary tumor [^18^F]FDG uptake levels for patient prognosis.

**Figure 6.**
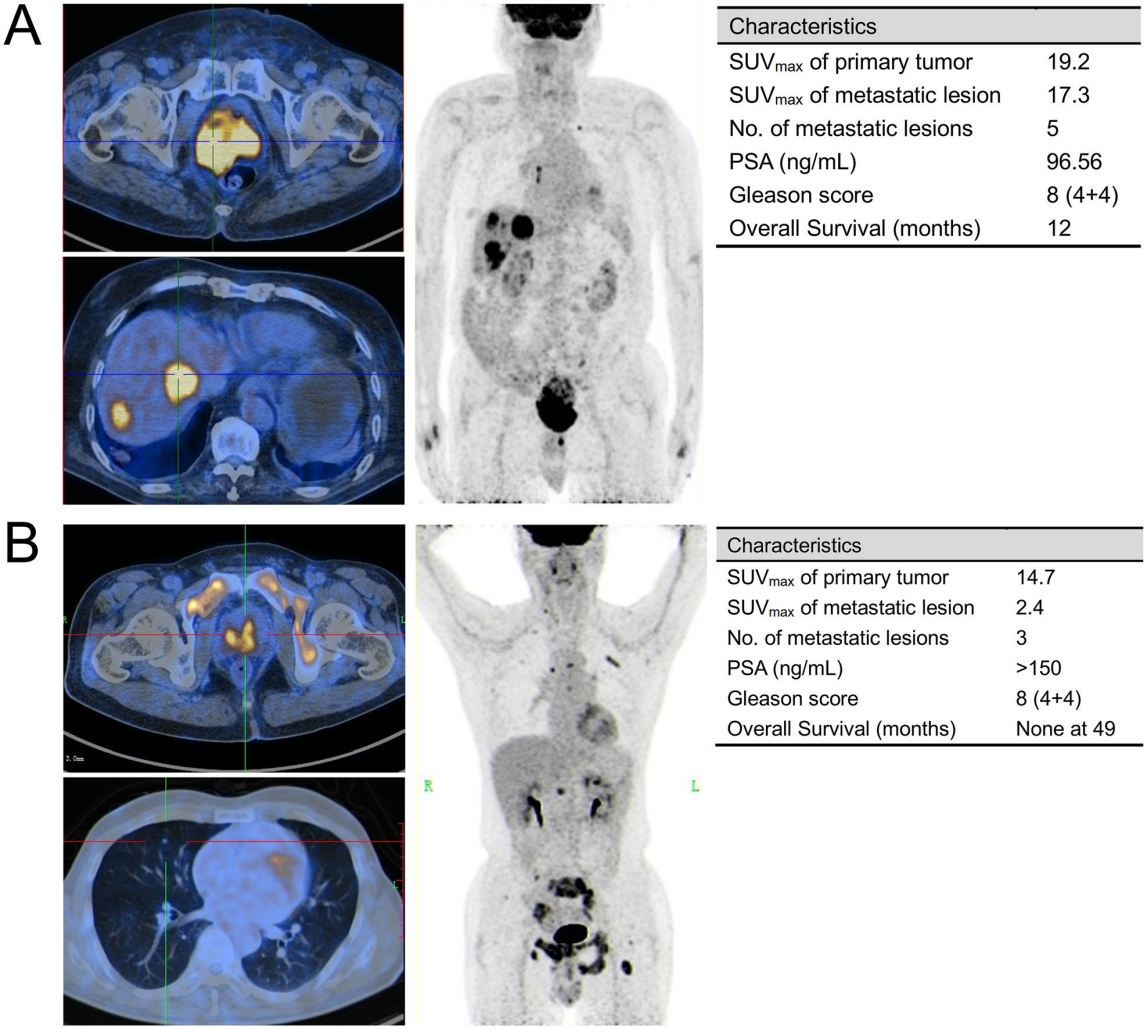
Two representative cases of mHSPC patients with similar baseline characteristics but distinct prognoses. (A) An 85-year-old man who was first diagnosed with liver metastatic prostate cancer in 2020, with Gleason score of 4 + 4 = 8 and serum PSA of 96.56 ng/ml. [^18^F]FDG PET/CT (upper left: axial PET/CT imaging of primary tumor; lower left: axial PET/CT imaging of liver metastatic lesion; Right: maximum intensity projection (MIP)) showed widespread visceral and lymph node metastasis. Although treated with endocrine therapy and chemotherapy immediately after diagnosis, he had a dismal prognosis, with an OS of 12 months from definitive PET/CT diagnosis to death; (B) a 67-year-old man who was first diagnosed with lung metastatic prostate cancer in 2018, with Gleason score of 4 + 4 = 8 and serum PSA of >150ng/ml. [^18^F]FDG PET/CT (upper left: axial PET/CT imaging of primary tumor; lower left: axial PET/CT imaging of lung metastatic lesion; right: maximum intensity projection (MIP)) showed multiple visceral and bone metastasis. This patient received ADT (including surgical castration) after being diagnosed. No evidence of tumor progression was noted with follow-up as of November 2022.

Figure 6 presented two representative cases alongside a summary of their clinical and imaging attributes. It indicated that the variations of [^18^F]FDG uptake in metastatic lesions profoundly influenced prognosis, even when such patients share identical PSA levels, Gleason Scores, and primary tumor imaging findings.

### Prediction of poor prognosis in patients with visceral and bone metastasis

Univariable Cox-regression analysis identified several factors significantly associated with poor OS in patients with visceral and bone metastases, as detailed in Tables 3 and 4, respectively. For visceral metastases, higher age (HR = 2.187, *p* = 0.035), liver metastasis (HR = 2.609, *p* = 0.014), and higher uptake of [^18^F]FDG in metastatic lesions (HR = 3.150, *p* = 0.003) were critical. While for bone metastases, only higher uptake of [^18^F]FDG in metastatic lesions (HR = 5.340, *p* < 0.001) served as a vital factor for poor OS. Similar factors have also been shown to significantly shorter PFS (Supplementary Tables 2 and 3). Liver metastasis (HR = 2.072, *p* = 0.028) combined with higher uptake of [^18^F]FDG in metastatic lesions (HR = 1.772, *p* = 0.043) were associated with an unfavorable PFS in patients with visceral metastases, while regional lymph node metastasis (HR = 1.676, *p* = 0.012) and higher uptake of [^18^F]FDG in metastatic lesions (HR = 2.406, *p* < 0.001) indicated a poorer PFS in patients with bone metastases.

**Table 3.** Prognostic factors for OS in patients with visceral metastases, selected by cox univariable and multivariable analysis.

Variants	Number	Prognostic factors for OS[1]			
		Univariable analysis		Multivariable analysis	
		HR[2] (95%CI[3])	*p*-Value	HR (95%CI)	*p*-Value
Age					
Lower than median age (<71)	43	Ref.	–	Ref.	–
Higher than median age (> =71)	47	2.187 (1.057-4.524)	*0.035	2.754 (1.259-6.024)	*0.011
Site of visceral metastasis					
Lung	61	Ref.	–	Ref.	–
Liver	21	2.609 (1.217-5.597)	*0.014	2.418 (1.106-5.289)	*0.027
Both lung and liver	8	2.465 (0.826-7.353)	0.11	2.528 (0.840-7.608)	0.099
Clinical T stage					
T = 2	29	Ref.	–		
T = 3 or 4	61	1.451 (0.651-3.236)	0.36		
Clinical *N* stage					
*N* = 0	24	Ref.	–		
*N* = 1	66	0.718 (0.340-1.518)	0.39		
PSA level					
Lower than median PSA (<79.62)	37	Ref.	–		
Higher than median PSA (> =79.62)	38	1.180 (0.545-2.557)	0.67		
Missing	15	–	–		
Gleason score					
6-7	15	Ref.	–		
8-10	47	0.906 (0.334-2.460)	0.85		
Missing	28	–	–		
[18F]FDG uptake in primary tumor					
Lower than median SUVmax (<6.10)	44	Ref.	–		
Higher than median SUVmax (> =6.10)	46	1.140 (0.567-2.296)	0.71		
[18F]FDG uptake in metastatic lesion					
Lower than median SUVmax (<2.85)	45	Ref.	–	Ref.	–
Higher than median SUVmax (> =2.85)	45	3.150 (1.485-6.682)	**0.003	4.399 (1.984-9.753)	***<0.001

[1] OS: overall survival.

[2] HR: hazard ratio.

[3] 95% CI: 95% confidence interval.

[4] Significance level set at p < 0.05. *p < 0.05, **p < 0.01, ***p < 0.001.

**Table 4.** Prognostic factors for OS in patients with bone metastases, Selected by Cox Univariable and Multivariable analysis.

Variants	Number	Prognostic factors for OS[1]			
		Univariable analysis		Multivariable analysis	
		HR[2] (95%CI[3])	*p*-Value	HR (95%CI)	*p*-Value
Age					
Lower than median age (<72)	81	Ref.	–		
Higher than median age (> =72)	96	0.644 (0.334-1.243)	0.188		
Clinical T stage					
T = 2	104	Ref.	–		
T = 3 or 4	73	1.707 (0.887-3.283)	0.109		
Clinical *N* stage					
*N* = 0	65	Ref.	–		
*N* = 1	112	1.859 (0.896-3.860)	0.096		
PSA level					
Lower than median PSA (<63.61)	71	Ref.	–		
Higher than median PSA (> =63.61)	71	1.873 (0.897-3.911)	0.095		
Missing	35	–	–		
Gleason score					
6-7	50	Ref.	–		
8-10	82	1.429 (0.592-3.446)	0.427		
Missing	45	–	–		
[18F]FDG uptake in primary tumor					
Lower than median SUVmax (<4.50)	87	Ref.	–		
Higher than median SUVmax (> =4.50)	90	1.010 (0.524-1.946)	0.976		
[18F]FDG uptake in metastatic lesion					
Lower than median SUVmax (<5.50)	86	Ref.	–	Ref.	–
Higher than median SUVmax (> =5.50)	91	5.340 (2.219-12.849)	***<0.001	5.340 (2.219-12.849)	***<0.001

[1] OS: overall survival.

[2] HR: hazard ratio.

[3] 95% CI: 95% confidence interval.

[4] Significance level set at p < 0.05. *p < 0.05, **p < 0.01, ***p < 0.001.

Multivariate Cox regression analysis confirmed that in patients with visceral metastases, higher age (HR = 2.754, *p* = 0.011), liver metastases (HR = 2.418, *p* = 0.027) and higher [^18^F]FDG uptake in metastatic lesions (HR = 4.399, *p* < 0.001) were significant independent prognostic indicators for OS, while liver metastasis (HR = 2.031, *p* = 0.034) was the sole prognostic indicator for poorer PFS. In patients with bone metastases, higher [^18^F]FDG uptake in the metastatic lesions (HR = 5.340, *p* < 0.001) was the only factor contributing to poorer OS. Additionally, for PFS, both regional lymph node metastasis (HR = 1.676, *p* = 0.012) and high [^18^F]FDG uptake in bone lesions (HR = 4.868, *p* < 0.001) were significantly associated with outcomes.

## Discussion

In our retrospective study, we analyzed the clinical characteristics and [^18^F]FDG PET/CT parameters of mHSPC patients, focusing on differentiating between visceral and bone metastasis characteristics and their prognostic implications. Our findings suggest that the SUV_max_ of [^18^F]FDG in metastatic lesions is a more effective indicator for OS and PFS than [^18^F]FDG uptake in primary tumors. This observation holds particularly true for both visceral and bone metastases, where higher [^18^F]FDG uptake correlates significantly with poorer survival outcomes. Our results also indicate that patients with bone metastases generally exhibit a better prognosis compared those with visceral metastases, which align with earlier clinical studies [7]. This underscores the critical need for precise and early detection strategies in visceral metastasis [15].

[^18^F]FDG PET/CT still faces significant challenges in the management of prostate cancer. Current clinical guidelines do not broadly recommend [^18^F]FDG PET/CT for routine management of prostate cancer, largely due to its reduced sensitivity to small liver metastases and challenges in detecting sub-centimeter lung metastases due to continuous lung motion [16,17]. Despite these challenges, our findings emphasize that [^18^F]FDG PET/CT significantly enhances prognostic assessments, particularly through the SUV_max_ of [^18^F]FDG in metastatic lesions as a predictive factor for poor survival. Building on previous research and earlier studies by our team, it has been observed that as prostate cancer progresses, there may be an increase in [^18^F]FDG uptake, potentially indicating neuroendocrine differentiation [13,18]. While this correlation suggests more aggressive tumor behavior and poorer outcomes, it underscores the strong prognostic value of [^18^F]FDG uptake in metastatic lesions, highlighting its importance in guiding clinical decision-making and tailoring treatment strategies for metastatic prostate cancer.

In fact, there is a compelling need for enhanced focus on visceral metastasis in the clinical management of prostate cancer, due to the challenge of early detection and intervention compared to bone metastasis, which significantly affects patients’ prognosis [7,19]. Consequently, our study aimed to shed light on this issue related to visceral metastasis in mHSPC. A previous retrospective study reported that 60% (932/1579) of identified visceral metastases in metastatic prostate cancer patients were lung lesions [20]. This aligns with other research indicating a more favorable prognosis for patients with lung metastases compared to those with liver or brain metastases [7,21–23]. Our findings corroborate these observations. Importantly, we identified liver metastasis as an independent predictor of poor prognosis. While liver metastasis has a reported incidence of just 0.3% among prostate cancer patients, in our cohort of patients initially diagnosed with metastatic prostate cancer, the incidence rose to 6.5% (29/447) [24]. This emphasizes the need for prompt diagnosis and treatment. No significant survival difference was observed between patients with combined lung and liver metastases compared to those with only lung metastases, a result possibly influenced by the small sample size of the former group.

This study, being retrospective in nature, has inherent limitations. We did not include [^68^Ga]Ga-PSMA-11 PET in our study due to insufficient data for analysis, despite its increasing clinical use. This exclusion limits us to directly compare its prognostic value against [^18^F]FDG PET. Our study also excluded mHSPC patients with visceral metastases in organs other than the lungs or liver, such as distant lymph nodes, brain or pancreas. Despite these limitations, our results advocate for the incorporation of advanced PET/CT modalities, including [^18^F]FDG, in routine clinical practice to refine the management of mHSPC, thereby enabling more targeted and effective therapeutic interventions. Future prospective studies on PET/CT with [^18^F]FDG and other tracers, using larger mHSPC cohorts, are anticipated to provide deeper insights and aid in refining diagnostic and treatment strategies.

## Conclusions

In our real-world analysis, we evaluated the prognostic significance of [^18^F]FDG SUV_max_ in metastatic lesions of mHSPC patients. Our results indicate that higher [^18^F]FDG uptake in metastatic lesions is associated with significantly shorter survival times and a higher likelihood of disease progression. Conversely, the [^18^F]FDG uptake in primary tumors did not demonstrate significant prognostic value. Furthermore, we identified that higher age and liver metastasis as significant risk factors for reduced survival in patients with visceral metastases. With the rising incidence of mHSPC, our study emphasizes the prognostic potential of [^18^F]FDG PET/CT in enhancing the initial diagnostic and treatment planning for prostate cancer.

## Ethical approval

The study was performed in line with the principles of the 1975 Declaration of Helsinki. Approval was granted by the Shanghai Jiao Tong University School of Medicine, Ren Ji Hospital Ethics Committee (RA-2021-242). The study received a waiver of informed consent from the Shanghai Jiao Tong University School of Medicine, Ren Ji Hospital Ethics Committee, due to the retrospective nature of the study and the use of anonymized clinical data.

## Supplementary Material

Revised supplementary tables.xlsx

## Data Availability

The datasets generated and analyzed during the current study are available from the corresponding author on reasonable request.
